# Adherence to Blended or Face-to-Face Smoking Cessation Treatment and Predictors of Adherence: Randomized Controlled Trial

**DOI:** 10.2196/17207

**Published:** 2020-07-23

**Authors:** Lutz Siemer, Marjolein G J Brusse-Keizer, Marloes G Postel, Somaya Ben Allouch, Robbert Sanderman, Marcel E Pieterse

**Affiliations:** 1 Technology, Health & Care Research Group Saxion University of Applied Sciences Enschede Netherlands; 2 Centre for eHealth and Well-being Research University of Twente Enschede Netherlands; 3 Medical School Twente Medisch Spectrum Twente Enschede Netherlands; 4 Department of Psychology, Health & Technology University of Twente Enschede Netherlands; 5 Tactus Addiction Treatment Enschede Netherlands; 6 Digital Life Research Group Amsterdam University of Applied Science Amsterdam Netherlands; 7 Department of Health Psychology University Medical Center Groningen Groningen Netherlands

**Keywords:** blended treatment, smoking cessation, adherence, predictors, tobacco, prevention

## Abstract

**Background:**

Blended face-to-face and web-based treatment is a promising way to deliver smoking cessation treatment. Since adherence has been shown to be an indicator of treatment acceptability and a determinant for effectiveness, we explored and compared adherence and predictors of adherence to blended and face-to-face alone smoking cessation treatments with similar content and intensity.

**Objective:**

The objectives of this study were (1) to compare adherence to a blended smoking cessation treatment with adherence to a face-to-face treatment; (2) to compare adherence within the blended treatment to its face-to-face mode and web mode; and (3) to determine baseline predictors of adherence to both treatments as well as (4) the predictors to both modes of the blended treatment.

**Methods:**

We calculated the total duration of treatment exposure for patients (N=292) of a Dutch outpatient smoking cessation clinic who were randomly assigned either to the blended smoking cessation treatment (n=130) or to a face-to-face treatment with identical components (n=162). For both treatments (blended and face-to-face) and for the two modes of delivery within the blended treatment (face-to-face vs web mode), adherence levels (ie, treatment time) were compared and the predictors of adherence were identified within 33 demographic, smoking-related, and health-related patient characteristics.

**Results:**

We found no significant difference in adherence between the blended and the face-to-face treatments. Participants in the blended treatment group spent an average of 246 minutes in treatment (median 106.7% of intended treatment time, IQR 150%-355%) and participants in the face-to-face group spent 238 minutes (median 103.3% of intended treatment time, IQR 150%-330%). Within the blended group, adherence to the face-to-face mode was twice as high as that to the web mode. Participants in the blended group spent an average of 198 minutes (SD 120) in face-to-face mode (152% of the intended treatment time) and 75 minutes (SD 53) in web mode (75% of the intended treatment time). Higher age was the only characteristic consistently found to uniquely predict higher adherence in both the blended and face-to-face groups. For the face-to-face group, more social support for smoking cessation was also predictive of higher adherence. The variability in adherence explained by these predictors was rather low (blended R^2^=0.049; face-to-face R^2^=0.076). Within the blended group, living without children predicted higher adherence to the face-to-face mode (R^2^=0.034), independent of age. Higher adherence to the web mode of the blended treatment was predicted by a combination of an extrinsic motivation to quit, a less negative attitude toward quitting, and less health complaints (R^2^=0.164).

**Conclusions:**

This study represents one of the first attempts to thoroughly compare adherence and predictors of adherence of a blended smoking cessation treatment to an equivalent face-to-face treatment. Interestingly, although the overall adherence to both treatments appeared to be high, adherence within the blended treatment was much higher for the face-to-face mode than for the web mode. This supports the idea that in blended treatment, one mode of delivery can compensate for the weaknesses of the other. Higher age was found to be a common predictor of adherence to the treatments. The low variance in adherence predicted by the characteristics examined in this study suggests that other variables such as provider-related health system factors and time-varying patient characteristics should be explored in future research.

**Trial Registration:**

Netherlands Trial Register NTR5113; http://www.trialregister.nl/trialreg/admin/rctview.asp?TC=5113

## Introduction

### Background

As smoking remains the leading cause of preventable death, cessation treatment is pivotal for public health promotion [1]. The introduction of electronic health (eHealth) [2] represents the expectation of information and communication technologies to improve health care [3]. However, adherence is generally low in web-based treatment [4] as well as in cessation treatment in general [5]. Low adherence is problematic because adherence has been shown to be an indicator of a treatment’s acceptability and a determinant of treatment effectiveness [6-9]. Therefore, adherence should be optimized because—assuming a dose-response relationship [10]—patients are more likely to quit smoking if they are more exposed to active ingredients of the treatment [6]. Adherence in general can be defined as the extent to which a person’s behavior (eg, taking medication, following a diet, or executing lifestyle changes) corresponds with recommendations from a health care provider [6]. In the context of behavioral change treatments such as smoking cessation, adherence issues are mainly related to premature termination of treatment and failure to perform tasks and exercises between sessions [11].

### Blended Treatment

In the past few decades, a variety of effective interventions for smoking cessation have become available [12,13], including more recently developed eHealth services such as web-based interventions [14,15] or mobile phone interventions [16,17]. At present, traditional face-to-face interventions on the one hand, and both web-based and mobile phone interventions on the other hand are increasingly being transferred to blended treatment. Blended treatment is a promising eHealth service, because it is expected that the strengths of one mode of delivery will compensate for the weaknesses of the other [4,18-23]. The main strength of face-to-face treatment is to provide personal attention of a professional, which could compensate for the lack of face-to-face contact in web-based treatment. In turn, a main feature of web-based care is the accessibility anytime and anywhere, which could compensate for the time between face-to-face sessions when the user needs support. Blended treatment is applied in diverse settings (eg, individual vs group setting [24]), addresses several health issues (eg, depression [25], anxiety [26], or addiction [10,22]), uses various tools (eg, web platforms, email, SMS text messaging, apps [20,27]), and uses different modes of delivery (eg, mainly web-based [28,29] vs mainly face-to-face [26,30] or integrated [10,22] vs sequential [29]). Since a clear definition of blended interventions is still missing [18], in this paper we define blended treatment as a combination of face-to-face sessions and web-based sessions to an integrated treatment that can be delivered by health care professionals on an outpatient basis. The blended intervention adopted in this study is an integrated equal blend of face-to-face treatment and treatment via an online platform.

### Adherence to Blended Treatment

Blended treatment has been shown to positively influence adherence [4,31-33]. However, to the best of our knowledge, no studies to date have directly compared adherence to a blended treatment with adherence to either a web-based treatment or a face-to-face treatment with identical active components. In this study, we used data from the LiveSmokefree study [22], which is a randomized controlled trial (RCT) comparing the effectiveness of a blended face-to-face and web-based smoking cessation treatment to a comparable face-to-face treatment. In a prior study, we explored measurement methods and levels and predictors of adherence to the blended treatment by including the blended treatment group participants of the RCT only [10]. In the current study, we extended this previous work by including participants from the face-to-face treatment, allowing for a direct comparison of the levels of adherence between blended and face-to-face treatments. Furthermore, to explore whether both modes of delivery within the blended treatment were used in equal frequency, we also focused on levels of adherence within the blended group in the two modes.

### Predictors of Adherence

When adherence is low, adherence predictors become an area of interest because they may provide insight into the cause of low adherence and can help to generate new approaches to improving treatment or better alignment between the patient and treatment. Adherence, in general, is determined by provider behaviors, health system factors, and patient characteristics, and the latter have been most extensively examined as predictors of adherence to traditional interventions [6]. Within the context of smoking cessation treatment—including both face-to-face and web-based treatments—several demographic, smoking-related, and health-related predictors of adherence have been examined. To date, several studies have indicated that the likelihood of being adherent may increase with a higher age [34,35], male gender [35], higher internet skills [36,37], negative attitude toward smoking and higher motivation to quit at baseline [38,39], higher self-efficacy at baseline [39], early success in quitting after the start of the treatment [7,34,40], and lower nicotine dependency at baseline with fewer withdrawal symptoms after quitting [35,38]. For blended treatment, our previous study showed that higher adherence was best predicted by marital status (ie, having a partner) and social modeling (ie, more nonsmoking friends/partner) [10]. Building on this work, we have expanded on these previous findings in the current study by examining the predictors for adherence to both treatment arms (blended and face-to-face) and additionally the two modes of delivery (face-to-face mode and web mode) in the blended arm.

### Objectives

In detail, this study explored the following questions. With respect to adherence, we asked (1) how adherent are participants to blended compared to face-to-face treatment? and (2) within the blended treatment group, how adherent are the participants to the face-to-face mode compared to the web mode? With respect to predictors, we asked (1) which demographic, smoking-related, and health-related patient characteristics predict adherence to blended and to face-to-face treatments, and to both groups combined? and (2) within the blended group, which of these characteristics predict adherence to the face-to-face mode and to the web mode?

## Methods

### Study Subjects

In this study, we used the already available data from patients (blended n=130; face-to-face n=162) of a not yet completed nonblinded RCT on the effectiveness of a blended smoking cessation treatment compared with a face-to-face treatment (LiveSmokefree study, n=172 allocated per group to determine a difference in abstinence rates of 5 percentage points with a power of 80% and =.025) [22]. The patients were referred to the outpatient smoking cessation clinic at Medical Spectrum Twente hospital (Enschede, the Netherlands) by the treating physicians of the hospital or by their general practitioners, and attended the initial treatment session between May 2015 and September 2018. Inclusion criteria were: (1) willing to quit smoking, (2) aged 18 or older, and (3) current daily smoker (at least one cigarette a day). Exclusion criteria were: (1) no internet access (ie, email, websites) and (2) not able to read or write in the Dutch language. In line with the Dutch Medical Research Ethics Committee guidelines, the study was approved by the accredited MEC Twente (P14-37/NL50944.044.14). Before initiation, the study was registered in the Netherlands Trial Registry (NTR5113). All patients had to sign an informed consent form before they were randomized.

### Randomization

Patients were randomly assigned to either the blended or face-to-face group. Randomization was performed at the individual level (allocation ratio 1:1) using QMinim Online Minimization [41]. The minimization was stratified according to: (1) level of internet skills [42], (2) level of nicotine dependence (Fagerstrom) [43,44], and (3) the quitting strategy favored by the patient (stop at once, gradual change, scheduled reduced smoking; for details see the description of the study intervention below). The data used for QMinim minimization were collected using the baseline questionnaire completed online by the patient at home prior to the start of treatment.

### Study Interventions

The study interventions to be compared were a blended face-to-face and web-based smoking cessation treatment and a face-to-face treatment alone. Except for the differences in mode of delivery (ie, face-to-face mode and web mode), both treatments included the following same features: (1) high-intensity treatments comprising 10 sessions with a total treatment time of 230 minutes (20 minutes each, except for the first that was 50 minutes); (2) delivered by health care professionals in an outpatient cessation clinic; (3) derived from the Dutch Guideline for Tobacco Addiction [45] fulfilling the requirements of the Dutch care module for smoking cessation [46]; (4) executed by counselors registered in the Dutch quality register of qualified smoking cessation counselors; (5) treatment costs reimbursed by the patient’s health insurance; (6) supporting three quitting strategies that were chosen at the start of the treatment (stop at once, change gradually by increasing the number of daily activities that are performed smoke-free, or decrease smoking at regular intervals such as scheduled smoking reduction 100%-75%, 75%-50%, etc). The chosen quitting strategy did not influence the course of the treatment in general. The order, pace, duration, and intensity were the same for all strategies.

Both the blended and face-to-face treatments included the following behavior change techniques, according to BCT taxonomy v1 of Michie et al [47]: 1.1 Goal setting (behavior), 1.2 Problem solving, 1.3 Goal setting (outcome), 1.4 Action planning, 1.5 Review behavior goal(s), 1.6 Discrepancy between current behavior and goal, 1.8 Behavioral contract, 1.9 Commitment, 2.3 Self-monitoring of behavior, 2.4 Self-monitoring of outcome(s) of behavior, 2.6 Biofeedback, 2.7 Feedback on outcome(s) of behavior, 3.1 Social support (unspecified), 4.2 Information about antecedents, 4.3 Reattribution, 5.1 Information about health consequences, 5.2 Salience of consequences, 5.3 Information about social and environmental consequences, 5.4 Monitoring of emotional consequences, 5.5 Anticipated regret, 5.6 Information about emotional consequences, 6.2 Social comparison, 6.3 Information about others’ approval, 7.4 Remove access to the reward, 8.1 Behavioral practice/rehearsal, 8.2 Behavior substitution, 8.3 Habit formation, 8.4 Habit reversal, 8.6 Generalization of a target behavior, 8.7 Graded tasks, 9.1 Credible source, 9.2 Pros and cons, 9.3 Comparative imagining of future outcomes, 10.7 Self-incentive, 10.9 Self-reward, 11.1 Pharmacological support (eg, nicotine replacement therapy [patches, gum], bupropion, varenicline), 11.2 Reduce negative emotions, 12.1 Restructuring the physical environment, 12.2 Restructuring the social environment, 12.3 Avoidance/reducing exposure to cues for the behavior, 12.4 Distraction, 13.1 Identification of self as role model, 13.2 Framing/reframing, 13.5 Identity associated with changed behavior, 14.4 Reward approximation, 14.5 Rewarding completion, 14.6 Situation-specific reward, 14.7 Reward incompatible behavior, 14.8 Reward alternative behavior, 15.1 Verbal persuasion about capability, 15.3 Focus on past success, and 16.3 Vicarious consequences.

The face-to-face treatment consisted of 10 face-to-face sessions delivered at the outpatient smoking cessation clinic. The blended treatment comprised 5 face-to-face sessions at the outpatient clinic and 5 web-mode sessions delivered via an online treatment platform. Both the face-to-face and blended treatments consisted of both counselor-dependent and counselor-independent components. The counselor-dependent web-based components of the blended treatment were interactive and relied on (asynchronous) communication (email, messaging) between the counselor and participant. The counselor-independent components such as psychoeducational content or a smoking diary were used by the participants on their own and in their own time. In the face-to-face group, these components were provided in a paper manual that the participants took home. In the blended treatment, these components were accessible online. As such, both treatments were equivalent with regard to content and intensity. An additional benefit of the blended treatment was that the content of previous counselor-dependent components remained accessible as email and messaging correspondence saved online.

The most characteristic feature of the blended treatment examined in this study is an equal balance between the face-to-face and web mode sessions; that is, the focus of the treatment is neither on face-to-face mode nor web mode. In addition, there is constant alternation and interactive use of the two modes. Table 1 shows the order, timing, main features, duration, and modes of delivery of the treatment sessions in the face-to-face and blended treatments. Although an equal distribution was planned for the blended treatment with regard to the number of sessions, there was an uneven distribution for the duration of treatment because the first session (50 minutes for face-to-face mode) was longer than the remaining sessions (20 minutes for the face-to-face mode or 20 minutes for web mode); therefore, the participants in the blended group spent 130 minutes in face-to-face mode and 100 minutes in web mode.

A detailed description of the treatments can also be found in the protocol article of the RCT [22] and in the description of the user experience of the blended smoking cessation treatment [48]. Screenshots of the web sessions of the blended treatment are shown in Multimedia Appendix 1 to provide an impression of the look and feel of the web interventions.

**Table 1:** Order, timing, main features, duration, and mode of delivery of the treatment sessions in the face-to-face and blended groups according to treatment protocol.

Session	Week	Main features	Duration (minutes)	Mode of delivery	
				BSCT^a^	F2F^b^
1	1	Goal setting; prompt smoking diary; measure CO^c^	50	F2F	F2F
2	3	Measures for self-control	20	Web	F2F
3	5	Dealing with withdrawal	20	F2F	F2F
4	7	Breaking habits	20	Web	F2F
5	9	Dealing with triggers	20	F2F	F2F
6	11	Food for thought	20	Web	F2F
7	14	Think differently; measure CO	20	F2F	F2F
8	18	Do differently	20	Web	F2F
9	22	Action plan; measure CO	20	F2F	F2F
10	26	Closure	20	Web	F2F

^a^BSCT: blended smoking cessation treatment; total duration=230 minutes (130 minutes F2F mode, 100 minutes web mode).

^b^F2F: face-to-face treatment; total duration=230 minutes.

^c^CO: carbon monoxide.

### Data Collection

#### Patient Characteristics

As part of the RCT (LiveSmokefree-study), 33 demographic, smoking-related, and health-related characteristics were assessed with the intake measurement using an online questionnaire. A detailed description of these characteristics is available in the protocol article of the RCT [22].

#### Measuring Adherence

Established measures for adherence to a blended treatment are still lacking. Therefore, for the 2018 study [10], we constructed a customized measure for adherence by selecting 18 patient activities (eg, using a web-based smoking diary tool, responding to counselors’ messages) to trace adherence to the blended treatment. Adequacy of this adherence measure was confirmed by the observed dose-response relationship between adherence and the likelihood of quitting, which is consistent with the smoking cessation literature [6-9]. However, this activity-based method was quite detailed and labor-intensive and was particularly interesting from a methodological point of view. Since the current study was mainly focused on comparing treatment modalities in a clinical context, we used a simpler time-based method for measuring adherence, which was proven to be as suitable for clinical research as the activity-based method and was also found to be more efficient [49,50]. Although this time-based method was not as accurate as the activity-based method, it was applicable in this case because the primary goal of this study was to determine differences between the groups in terms of levels and predictors of adherence. Therefore, the analysis of relative level differences was more relevant than an exact measurement of absolute levels. Furthermore, the time-based method allowed for analysis of a larger sample and thus more accurate statistics, as it required less time and money.

For this time-based approach, we used treatment data from the hospital’s electronic patient record system. This record system contains basic information of the patients’ treatment status such as when the patient started treatment; which counselor was offering the treatment; time, day, and type of appointments; time and day of telephone consults; and which kind of treatment was offered in each appointment [51]. In this record system, the counselors reported, in an encoded form, which type of sessions were completed. Each code represents a fixed, average number of minutes invested in face-to-face mode or web mode, as shown in Table 2. These fixed numbers of minutes per sessions were used to calculate the total number of minutes in treatment for each patient for the blended and face-to-face treatments, as well as for the face-to-face mode and web mode in the blended treatment.

**Table 2:** Codes, descriptions, modes of delivery, and duration of face-to-face (F2F) and blended treatment sessions used to measure adherence.

Code	Description of the session	Mode	Duration (minutes)
RSN	First individual F2F session at treatment start	F2F	50
RSAB	Like RSN, but visiting a patient at another department of the hospital	F2F	50
RSNS	Like RSN, but with 2 patients at the same time (eg, husband and wife)	F2F	35
RSC	Usual individual F2F session	F2F	20
RSAC	Additional consult (to add to RSN/RSAB/RSNS/RSC if more time is needed)	F2F	20
RSTC	Individual telephone consult	F2F	20
RSOC	Any other individual consult	F2F	10
RSIC	Web-mode treatment session via rokendebaas.nl	Web	20
RSEC	Email consulting	Web	10

### Statistical Analysis

All analyses were performed using SPSS version 24.

#### Patient Characteristics

For both the blended and face-to-face groups, 33 demographic, smoking-related, and health-related characteristics were measured and are reported as means (SDs) for normally distributed continuous variables and as medians (IQRs) for nonnormally distributed continuous variables. Categorical variables are reported as numbers with corresponding percentages. To identify between-group differences within the 33 demographic, smoking-related, and health-related patient characteristics, independent *t* tests or Mann-Whitney *U* tests were performed as appropriate for continuous variables; the Pearson Chi-square or Fisher exact test was performed for categorical variables.

#### Adherence (Time Spent in Treatment)

Based on the hospital administrational records, both the absolute treatment time (in minutes) and the proportional treatment time (in percentage) of the patients who had started treatment were calculated for the blended and face-to-face groups, as well as for the face-to-face mode and web mode of the blended treatment. Bar charts were used to compare how many patients spend how much time in the blended and face-to-face treatment on the one hand and in each mode of the blended treatment on the other hand. Mann Whitney *U* tests were performed to compare the absolute treatment time of the blended and face-to-face treatments and the proportional treatment time for the face-to-face and web modes in the blended treatment.

#### Predictors of Adherence

To identify the predictors of adherence (as a continuous variable) within the 33 demographic, smoking-related, and health-related patient characteristics, Pearson or Spearman correlation tests were performed as appropriate for continuous variables; independent *t* tests or Mann-Whitney *U* tests were performed for dichotomous variables. Variables with significance at *P*<.15 were considered as candidates for multivariate linear regression analyses. They were first tested with univariate linear regression analyses so that univariate and multivariate odds ratios could be compared, and were entered in the multivariate linear regression analyses after checking for multicollinearity. The variables were either all entered and removed step by step via the backward selection method (all patients; blended group; face-to-face group; face-to-face mode of the blended treatment) or entered step by step via the forward selection method (web mode of the blended treatment). Variables were entered or eliminated step by step based on the model fit. In the case of multicollinearity, the variable with the best model fit was selected for linear analyses.

## Results

### Participant Flow

**Figure 1:**
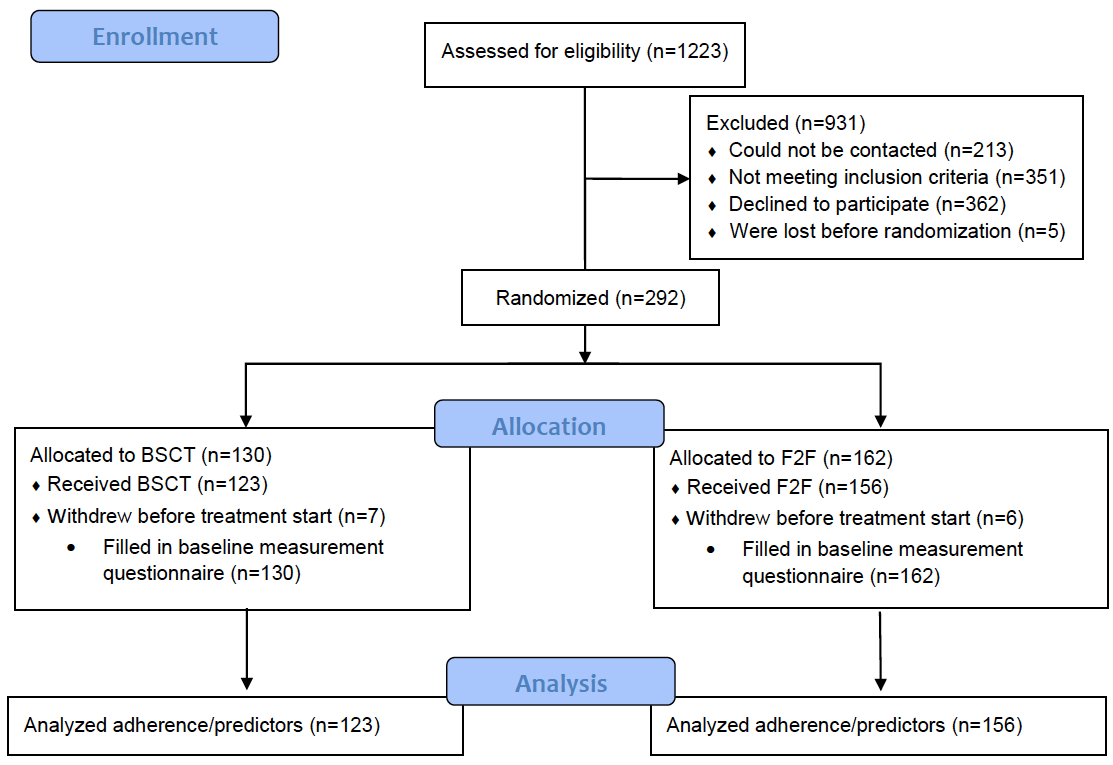
Flow of participants through the study. BSCT: blended smoking cessation treatment; F2F: face-to-face.

Figure 1 shows the flow of participants through the study. A total of 292 patients were eligible for the study, provided written consent, filled out the baseline questionnaire, and were randomized (blended n=130; face-to-face n=162). Before the start of treatment, 7/130 (5.4%) patients of the blended group and 6/162 (3.7%) patients of the face-to-face group withdrew. Finally, data from 123/130 (94.6%) patients in the blended group and 156/160 (96.3%) patients in the face-to-face group were available for adherence analysis.

### Patient Characteristics

Table 3 shows the patients’ characteristics for both the blended group (n=130) and the face-to-face group (n=162). Significant differences (*P*<.05) between the blended and face-to-face group were found for 6 of the 33 characteristics. Patients in the face-to-face group had higher internet skills, used more medication in general, reported less health complaints, and scored higher on the Depression Anxiety Stress Scale (DASS) subscales depression and anxiety, and on the total DASS score.

**Table 3:** Patients’ characteristics of both the blended smoking cessation treatment (BSCT) and face-to-face (F2F) groups.

Characteristic			BSCT (n=130)		F2F (n=162)		*P* value	
**Demographic characteristics**								
	Sex (female), n (%)		62 (47.7)		77 (47.5)		.98	
	Age (years), mean (SD)		47.1 (12.8)		46.6 (13.2)		.76	
	**Marital status, n (%)**							.18
		With partner	87 (66.9)		96 (59.3)			
		Single	43 (33.1)		66 (40.7)			
	**Housing situation, n (%)**							.91
		Children	54 (41.5)		65 (40.9)			
		No children	76 (58.5)		94 (59.1)			
	**Education, n (%)**							.88
		VET^a^ or higher	82 (63.1)		101 (63.9)			
		Lower than VET	48 (36.9)		57 (36.5)			
	**Main income, n (%)**							.73
		Wage or own company	64 (48.2)		83 (51.2)			
		Income support	66 (50.8)		79 (48.8)			
	**Main day activity, n (%)**							.72
		Paid work	61 (46.9)		79 (49.1)			
		Other	69 (53.1)		82 (50.9)			
	Internet skills^b^, mean (SD)		38.5 (5.64)		40.52 (8.63)		.01	
**Smoking-related characteristics**								
	**Reason to start treatment, n (%)**							.70
		Intrinsic	83 (63.8)		107 (66.0)			
		Extrinsic	47 (36.2)		55 (34.0)			
	Nicotine dependency^c^, mean (SD)		5.29 (2.10)		5.00 (2.18)		.59	
	Negative attitude toward quitting^d^, mean (SD)		–5.70 (3.16)		–5.00 (2.96)		.07	
	Positive attitude toward quitting^e^, median (IQR^c^)		10 (8-12)		10 (8.75-11)		.91	
	Self-efficacy^f^, mean (SD)		–0.37 (5.32)		–0.45 (5.02)		.89	
	Readiness to quit^g^, median (IQR)		2 (1-3)		2 (1-3)		.31	
	Earlier quit attempts, n (%)		108 (83.1)		143 (88.3)		.20	
	Social support^h^, median (IQR)		4 (3-5)		4 (3-5)		.99	
	Social modeling^i^, median (IQR)		3.5 (1-6)		3 (1-5)		.13	
	Use of alcohol^j^, n (%)		2 (1-3)		2 (0.75-3)		.26	
	Use of (recreational) drugs, n (%)		11 (8.5)		14 (8.7)		.94	
**Health-related characteristics**								
	Use of medication in general, n (%)		85 (65.4)		123 (75.9)		.05	
	Use of medication for addiction treatment, n (%)		0 (0.0)		0 (0.0)		N/A^k^	
	Use of medication for psychiatric treatment, n (%)		26 (20.0)		23 (15.1)		.28	
	Use of medication for physical treatment, n (%)		64 (49.2)		88 (57.9)		.15	
	Use of other medication, n (%)		19 (14.6)		31 (20.4)		.21	
	Health complaints (MAPHSS^l^), mean (SD)		12.58 (6.27)		10.96 (7.17)		.04	
	Smoking-related complaints^m^, mean (SD)		20.82 (9.17)		19.95 (8.86)		.41	
	Health and smoking-related complaints^n^, mean (SD)		33.56 (13.87)		30.91 (14.42)		.11	
	Depression^o^, median (IQR)		4 (0-10)		4 (2-24)		.02	
	Anxiety^o^, median (IQR)		4 (2-8)		6 (2-16.5)		.002	
	Stress^o^, median (IQR)		8 (4-16)		10 (4-14)		.73	
	DASS^p^, median (IQR)		18 (8-32)		22 (8-58.5)		.01	
	EQ-5D-3L^q^, median (IQR)		0.77 (0.69-1.00)		0.77 (0.69-1.00)		.42	
	EQ VAS^r^, mean (SD)		66.95 (16.88)		65.17 (17.56)		.38	

^a^VET: vocational education and training.

^b^Scored on a scale of 10-60; a higher score indicates better skills.

^c^Fagerstroem scale (range 0-10); a higher score indicates higher nicotine dependency.

^d^Scored on a scale of –12 to 0; a lower score indicates a more negative attitude.

^e^Scored on a scale of –12 to 0; a higher score indicates a more positive attitude.

^f^Scored on a scale of –12 to 12; a higher score indicates higher self-efficacy.

^g^Scored on a scale of 0-4; a higher score indicates greater readiness to quit.

^h^Scored on a scale of 0-5; a higher score indicates more social support.

^i^Scored on a scale of 0-8; a higher score indicates more smokers in the social environment.

^j^Scored on a scale of 0-4; a higher score indicates higher alcohol consumption.

^k^N/A: not applicable; no statistical analysis performed since the variable is constant.

^l^MAPHSS: Maudsley Addiction Profile Health Symptoms Scale (range 0-40; a higher score indicates poorer health status).

^m^Scored on a scale of 0-64; a higher score indicates more smoking-related complaints.

^n^Scored on a scale of 0-104; a higher score indicates poorer health status and more smoking-related complaints.

^o^Scored on a scale of 0-42; a higher score indicates a higher level of depression/anxiety/stress.

^p^DASS: Depression Anxiety Stress Scale; sum of the Depression, Anxiety, and Stress subscale scores (range 0-126; a higher score indicates a more negative emotional status).

^q^EQ-5D-3L: societal-based quantification of health status (range 0-1; a higher score indicates better health status).

^r^EQ VAS: visual analog scale for quality of life (range 0-100; a higher score indicates better health status).

### Adherence (Time Spent in Treatment)

**Figure 2:**
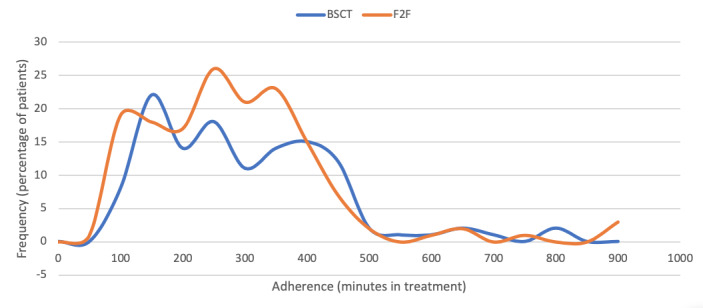
Adherence to blended smoking cessation treatment (BSCT) vs face-to-face (F2F) treatment.

**Figure 3:**
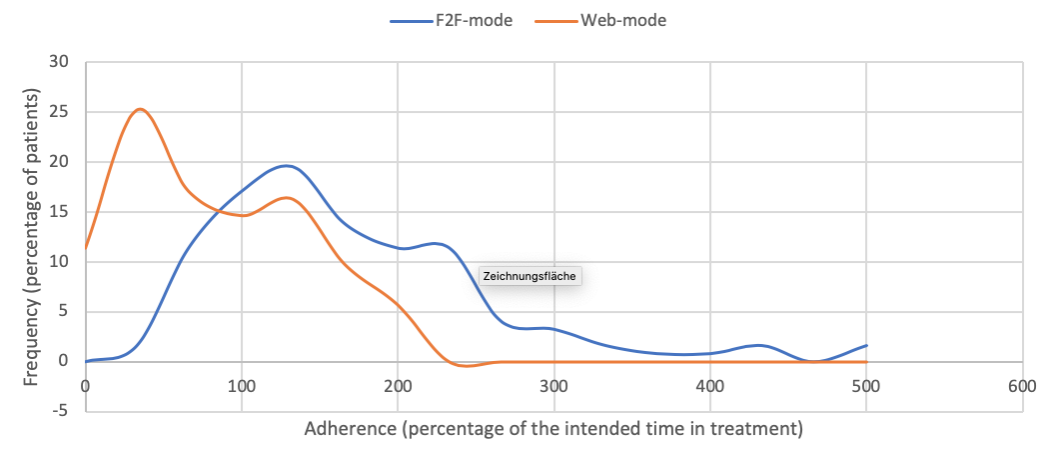
Adherence within the blended smoking cessation treatment group to the two modes of the treatment: face-to-face (F2F) mode vs web mode.

As illustrated in Figure 2, adherence to the blended and face-to-face treatments was comparable. Patients in the blended group (n=123, 7 patients dropped out between inclusion and the first treatment session) spent a median of 246 (IQR 150-355) minutes in treatment (106.7% of the intended total treatment time); in the face-to-face group (n=156, 6 patients dropped out between inclusion and first treatment session), the patients spent a median of 238 (IQR 150-330) minutes in treatment (103.3% of the intended total treatment time). There was no significant difference between the two groups (*P*=.30). However, within the blended group, as shown in Figure 3, patients were more adherent to the face-to-face mode than to the web mode. Patients in the blended group (n=123) spent a mean of 198 (SD 120) minutes in face-to-face mode and 75 (SD 53) minutes in web mode. In proportion to the intended treatment time for each mode of delivery (face-to-face mode=130 minutes; web mode=100 minutes), patients in the blended group spent twice the time in face-to-face mode (mean 152%, SD 92% of 130 minutes) than in web mode (mean 75%, SD 53% of 100 minutes) (*t*_122_=10.03; *P*<.001).

### Predictors of Adherence

For both treatments together, 7 predictors (Table 4) were significantly associated with higher adherence in the univariate analysis (assessed at *P*<.15), including male sex, older age, housing situation (living without children), higher readiness to quit, higher social support, lower social modeling (less smokers in the social environment), and higher use of other medication. Multivariate regression analyses (Table 5) revealed that age was the best predictor of adherence (R^2^=0.047). Per life year, patients spent 2.5 more minutes in treatment (95% CI 1.2-3.8; *P*=.001).

For the face-to-face group, 6 predictors (Table 4) were significantly associated with higher adherence in the univariate analyses (assessed at *P*<.15), including older age, higher readiness to quit, more social support, lower social modeling, higher use of other medication, and higher smoking-related complaints. Multivariate regression analyses (Table 5) revealed that age and social support together were the best predictors of adherence (R^2^=0.076). Per life year, patients spent 2.2 minutes more in treatment (95% CI 0.4-3.9; *P*=.02). For social support, graded from 0 (low social smoking cessation support) to 5 (high social smoking cessation support), each unit increase was associated with 20.5 more minutes in treatment (95% CI 2.3-38.8; *P*=.03).

For the blended group, 8 predictors (Table 4) were significantly associated with higher adherence in the univariate analyses (assessed at *P*<.15), including higher age, housing situation (living without children), lower nicotine dependency (Fagerstroem), higher negative attitude toward quitting, lower social modeling, lower health complaints, lower anxiety, and lower stress. Multivariate regression analyses (Table 5) revealed that age was the best predictor of adherence (*P*=.01). Per life year, patients spent 2.6 more minutes in treatment (95% CI 0.5-4.6; R^2^=0.049).

For the face-to-face mode of the blended treatment, 3 predictors (Table 6) were significantly associated with higher adherence in univariate analyses (assessed at *P*<.15), including higher age, housing situation (living without children), and lower internet skills (Table 6). Multivariate regression analyses (Table 7) revealed that housing situation was the best predictor of face-to-face mode adherence (R^2^=0.034). Patients living without children spent 49.7 more minutes in the face-to-face mode of the blended treatment (95% CI 92.7-6.8; *P*=.02) (Table 7).

For the web mode of the blended treatment, 16 predictors (Table 6) were significantly associated with higher adherence in univariate analyses (assessed at *P*<.15), including male sex, older age, main income (income support), main day activity (other than paid work), extrinsic reason to start treatment, lower nicotine dependency (Fagerstroem), higher negative attitude toward quitting, higher self-efficacy, lower social modeling, lower health complaints (assessed on the Maudsley Addiction Profile Health Symptoms Scale [MAPHSS]), lower smoking-related complaints, lower health and smoking-related complaints, lower anxiety, lower stress, lower DASS, and higher quality of life (EQ-5D-3L). Health and smoking-related complaints and the DASS were not used for multivariate regression because of multicollinearity. Multivariate regression analyses (Table 7) revealed that reason to start treatment, negative attitude toward quitting, and health complaints (MAPHSS) together were the best predictors of web mode adherence (R^2^=0.164). Patients with an intrinsic motivation spent 21.5 less minutes in the web mode of the blended treatment (95% CI –39.8 to –3.3; *P*=.02). For negative attitude toward quitting (range –12 to 0; lower numbers indicate a more negative attitude toward quitting smoking), each unit increase (ie, a less negative attitude) was associated with 3.6 more minutes in web mode of the blended treatment (95% CI 0.9-6.4, *P*=.01). For health complaints (range 0-40; higher numbers indicate poorer health status), each unit increase (ie, additional complaint reported) was associated with 2.4 less minutes in the web mode of the blended treatment (95% CI –3.8 to –1.0, *P*=.001).

**Table 4:** Univariate predictors for adherence in all patients and in each treatment group.

Characteristic		All patients			F2F^a^			BSCT^b^	
		Regression coefficient (95% CI)	*P* value	Regression coefficient (95% CI)		*P* value	Regression coefficient (95% CI)		*P* value
**Sex**			.11			—^c^			—
	Female (reference)	N/A^d^		—			—		
	Male	28.6 (–6.4-63.6)		—			—		
Age (years)		2.5 (1.2-3.8)	.001	2.4 (0.7-4.2)		.01	2.6 (0.5-4.6)		.01
**Housing situation**			.13			—			.05
	Children (reference)	N/A		—			—		
	No children	27.8 (–8.0-63.6)		—			52.9 (105.6-0.4)		
Nicotine dependency		—	—	—		—	–10.9 (–23.3-1.4)		.08
Negative attitude toward quitting		—	—	—		—	6.6 (–1.6-14.7)		.11
Readiness to quit		16.0 (–1.5-33.6)	.07	21.1 (–3.3-45.5)		.09	—		—
Social support		13.6 (0.5-26.8)	.04	23.3 (4.5-41.7)		.01	—		—
Social modeling		–8.4 (–15.5 to –1.5)	.02	–10.1 (–20.0 to –0.1)		.05	–7.5 (–17.1-2.2)		.13
**Use of other medication**			.06			.06			—
	Yes (reference)	N/A		N/A			—		
	No	–36.8 (–75.0-1.3)		–53.2 (–108.1-1.7)			—		
Health complaints (MAPHSS^e^)		—		—			–3.4 (–7.5-0.7)		.10
Smoking-related complaints		—		2.20 (–0.5-4.9)		.10	—		
Anxiety		—		—			–3.5 (–8.0-0.9)		.12
Stress		—		—			–2.8 (–6.2-0.6)		.11

^a^F2F: face-to-face treatment group.

^b^BSCT: blended smoking cessation treatment group.

^c^Data not shown, since for the sake of clarity only variables included in the multivariate regression at *P*<.15 are shown in the table.

^d^N/A: not applicable.

^e^MAPHSS: Maudsley Addiction Profile Health Symptoms Scale.

**Table 5:** Multivariate model of patient characteristics predicting adherence for all patients and each treatment group.

Variable	All patients		F2F^a^		BSCT^b^	
	Regression coefficient (95% CI)	*P* value	Regression coefficient (95% CI)	*P* value	Regression coefficient (95% CI)	*P* value
Age (years)	2.5 (1.2-3.8)	.001	2.2 (0.4-3.9)	.02	2.6 (0.5-4.6)	.01
Social support	—^c^	—	20.5 (2.3-38.8)	.03	—	—

^a^F2F: face-to-face treatment.

^b^BSCT: blended smoking cessation treatment.

^c^Data not shown, as for the sake of clarity only the variables of the final models are presented here.

**Table 6:** Univariate predictors for adherence to face-to-face (F2F) mode and web mode in the blended treatment group.

Variable		F2F mode		Web mode	
		Regression coefficient (95% CI)	*P* value	Regression Coefficient (95% CI)	*P* value
**Sex**					.12
	Female (reference)	N/A^a^		N/A	
	Male	—^b^		14.9 (33.8 to –3.9)	
Age		1.9 (0.2-3.6)	.03	0.8 (0.1-1.5)	.03
**Housing situation**			.02		—
	Children (reference)	N/A		—	
	No children	49.7 (6.8 - 92.7)		—	
**Main income**			—		.07
	Wage or own company (reference)	—		N/A	
	Income support	—		–17.2 (–36.0 to –1.5)	
**Main day activity**			—		.02
	Paid work (reference)	—		Ref	
	Other	—		–21.7 (–40.3 to –3.1)	
Internet skills		–3.0 (–6.8-0.8)	.12	—	—
**Reason to start treatment**			—		.09
	Extrinsic (reference)	—		N/A	
	Intrinsic	—		–16.9 (–36.4-2.5)	
Nicotine dependency		—	—	–6.1 (–10.5 to –1.8)	.01
Negative attitude towards quitting		—	—	3.8 (0.9-6.7)	.01
Self-efficacy		—	—	1.4 (–0.3-3.2)	.12
Social modeling		—	—	–3.1 (–6.6-0.4)	.08
Health complaints (MAPHSS^c^)		—	—	–2.4 (–3.8 to –0.9)	.001
Smoking-related complaints		—	—	–1.0 (–2.0-0.1)	.06
Health and smoking-related complaints		—	—	–0.9 (–1.6 to –0.3)	.01
Anxiety		—	—	–2.2 (–3.7 to –0.6)	.01
Stress		—	—	–1.4 (–2.6 to –0.2)	.03
DASS^d^		—	—	–0.6 (–1.1 to –0.1)	.02
EQ-5D-3L^e^		—	—	28.9 (–8.1-65.8)	.13

^a^N/A: not applicable.

^b^Data not shown, since for the sake of clarity only variables that were included in the multivariate regression at *P*<.15 are shown in the table.

^c^MAPHASS: Maudsley Addiction Profile Health Symptoms Scale.

^d^DASS: Sum of Depression, Anxiety, and Stress scores.

^e^EQ-5D-3L: societal-based quantification of health status.

**Table 7:** Multivariate model of patient characteristics predicting adherence to face-to-face (F2F) and web mode in the blended treatment group.

Variable		F2F mode				Web mode		
		Regression coefficient (95% CI)		*P* value		Regression coefficient (95% CI)		*P* value
**Housing situation**				.02				—^a^
	Children (reference)		N/A^b^				—	
	No children		49.7 (6.8-92.7)				—	
**Reason to start treatment**				—				.02
	Extrinsic (reference)		—				N/A	
	Intrinsic		—				–21.5 (–39.8 to –3.3)	
Negative attitude toward quitting			—		—		3.6 (0.9-6.4)	.01
Health complaints (MAPHSS^c^)			—		—		–2.4 (–3.8 to –1.0)	.001

^a^Data not shown, as for the sake of clarity only the variables of the final models are presented here.

^b^N/A: not applicable.

^c^MAPHSS: Maudsley Addiction Profile Health Symptoms Scale.

## Discussion

### Principal Findings

Since the emergence of web-based health promotion counseling a few decades ago, blended treatments have recently been introduced. The aim of the present study was to directly compare adherence to a blended treatment with a face-to-face treatment for smoking cessation with similar content.

Based on the treatment times documented in the hospital administration, we found comparable adherence levels for the blended and face-to-face treatments. However, within the blended treatment, we found that patients spent twice as much time in face-to-face mode (152% of the intended treatment time) than in web mode (75% of the intended treatment time), suggesting a tendency to substitute web sessions by additional face-to-face sessions.

Older age was the only characteristic consistently found to predict higher adherence to both the face-to-face and blended treatments. For the face-to-face group, we found that both older age and perceived social support for smoking cessation predicted higher adherence. Age is known as a relevant demographic characteristic for predicting adherence [34,35], but more social support to quit smoking has not yet been indicated as an independent predictor of adherence.

Within the blended treatment, no consistent predictor of adherence was found for its two modes of delivery. Higher adherence to the face-to-face mode was predicted by the housing situation (ie, living without children), whereas adherence to the web mode was predicted by an extrinsic motivation to quit, a less negative attitude toward quitting, and less health complaints. Although these models contained statistically significant predictive patient characteristics, the predicted proportion of variability in adherence was small, ranging from 3.4% to 16.4%. Thus, it seems immature to interpret these findings in an attempt to understand the mechanisms in adherence to blended smoking cessation treatment, and it is difficult to find a meaningful pattern in these predictors. To explain this low model fit, two aspects can be considered. First, this could indicate that the predictors examined in this study, namely only the patient characteristics, are not comprehensive. For example, it seems likely that provider-related variables and health care system factors such as treatment costs, failure to recall a receipt of a prescription, and access to free nicotine replacement therapy [6] also play a role. As no data on these factors were available in this study, this could not be further verified. Second, all patient-related predictors used in the current study were evaluated at the start of treatment, which means that changes in these characteristics during treatment (eg, due to negative treatment effects such as weight gain, adverse events, or withdrawal symptoms) were not considered. As an example of a positive treatment effect, in the context of smoking cessation treatment, the bidirectional relation between quitting success and adherence is known, in which early quitting success predicts higher adherence [34], while higher adherence predicts (long-term) abstinence [6-9]. Another example is the user experience that patients build during the course of treatment. Patients may experience the treatment as “useful,” “easy to follow,” or “stimulating” and adhere to the treatment accordingly [48,52].

In general, the finding for the blended group that treatment time not used in web mode was compensated by face-to-face mode treatment would support the expectation that in blended treatment, the strengths of one mode of delivery will compensate for the weaknesses of the other [4,18-23]. This expectation is also supported based on our recently published qualitative study on user experience with this blended smoking cessation treatment [48,52], in which we also found that the strengths of the face-to-face mode can compensate for the weaknesses of the web mode. It is noteworthy that this compensation is mainly unidirectional: face-to-face mode compensates or replaces web mode and not vice versa. By exceeding the planned face-to-face treatment time by 100 minutes on average, the vast majority of patients in the blended group (118/123, 95.9%) spent significantly more time in face-to-face mode than in web mode. By contrast, only 5/123 (4.1%) patients spent slightly more time (an additional 27 minutes on average) in web mode than in face-to-face mode. Perhaps the new and challenging web mode is not used optimally, as it can (easily) be compensated by the traditional, familiar face-to-face mode.

Although the main objective of this study was to provide a treatment time–based comparison of adherence, we would like to briefly mention two aspects that surprised us when comparing the results with one of our previous studies [10] that used a different operationalization of adherence.

First, the current study revealed rather high adherence to both the blended and face-to-face treatments. Due to differences in interventions, measurements of adherence, adjunctive support, and investigated populations, adherence rates for smoking cessation treatment vary widely between different studies (5%-96%) [6]. This makes it difficult to compare adherence rates in general. Moreover, little is known about adherence rates for blended treatment. We only found one study that reported adherence rates: in a blended depression treatment, adherence to the blended treatment (90.5%) and the face-to-face treatment (95.1%) was comparable [53]. Our study seems to agree with this previous study, as we also found comparably high adherence to the blended (106.7%) and face-to-face (103.3%) treatments. Surprisingly, for the blended treatment, the findings in this study seem to contradict our findings from a previous study among participants of the blended treatment in the same sample [10], in which we reported that adherence to the blended treatment seemed rather low. These apparent contradictory results may be explained by different operationalizations of adherence and different measurement methods in the two studies. In the 2018 study, we traced treatment activities of the patients in detail (not only treatment time as used in the current study) and strived for a categorical threshold-based classification of patients as being either adherent or nonadherent. This activity-based method used in the 2018 study correlates with the time-based measurement applied in the current study, but it was more specific [49,50] and therefore resulted in lower absolute adherence rates [10].

Second, in our 2018 study [10], we found that in the blended treatment, based on patients’ activities, there was no significant difference in adherence to the face-to-face mode compared with the web mode. Surprisingly, in the current study, based on treatment time, the adherence levels differed significantly. Patients spent only 75.2% of the intended treatment time in the web mode, but 152.3% of the intended treatment time in the face-to-face mode. This shows that in practice it is rather a 2/3 to 1/3 ratio between face-to-face mode and web mode in the blended group than the planned equal ratio. This could mean, for example, that patients in face-to-face mode need more time than planned for their activities, or that additional unplanned activities take place within the treatment time. This could be an indication of therapist drift—a known weakness of face-to-face treatment [30]—and thus bring the topic of treatment fidelity into focus. From a clinical point of view, the question then arises as to whether the planned times for face-to-face mode and web mode are appropriate.

### Limitations and Implications for Future Work

To the best of our knowledge, this is the first study to compare adherence and predictors of adherence in patients randomly assigned to either a blended smoking cessation treatment or a face-to-face treatment with identical active components. Moreover, this is also the first study to compare adherence and predictors of adherence to the face-to-face mode with those to the web mode of a blended smoking cessation treatment. One limitation of this study is that the measurement of adherence was based on the treatment time documented in the hospital’s administrational records, as this documentation is mainly used for financial accounting and therefore does not reflect in detail the contents and the exact temporal proportions of the treatments. Even though we assume that we have a sufficiently valid measure for the comparison of adherence and for the determination of predictors, these data unfortunately do not provide deeper insight into the adherence to the treatment process in detail. For example, the specific treatment activities carried out in different time frames remain unclear. In addition, the time data for the sessions were standard values and not exactly determined as treatment time per session. Individual sessions may therefore have been shorter or longer than evaluated. The absolute time values should therefore be interpreted with caution. Furthermore, in view of the differences between the results of this study and those of the 2018 study [10], in which different operationalizations of adherence were applied (time-based vs activity-based), the methodological question also arises as to which operationalization best reflects adherence. From our previous studies [49,50], we know that activity-based measurement has better predictive validity, which makes it seem more adequate when adherence is considered a determinant of efficacy (dose-response relationship). In this study, however, we used a time-based measurement because it requires less financial and time effort, the possibility of analyzing a larger sample size allowed us to expect more accurate statistics, and because we wanted to gain more experience with its application in clinical practice. The differences found in adherence between the 2018 study and the present study bring forth an interesting issue that deserves more attention and should be targeted in future studies, such as by addressing the research questions of this study using activity-based measurement to analyze the entire sample.

Another limitation is the low variability of adherence explained by our prediction models. The question arises as to whether the chosen predictors and their measurement are sufficient. Future research should further investigate which additional predictors (eg, provider behavior, health system factors, or other patient characteristics) should be included and how these can be measured, not only at the beginning but also over the course of treatment, so that they fit optimally to the research question.

Another point of interest should be the difference in predictors and levels of adherence to the two modes of delivery in the blended treatment. The characteristics associated with adherence are quite different (adherence to the face-to-face mode of the blended treatment was mostly associated with demographic characteristics, whereas adherence to the web mode of the blended treatment was mainly associated with smoking-related and health-related characteristics). Future research should examine the causation of these differences. For example, it is possible that web-mode treatment is better suited for patients with less health complaints because they rely less on a hospital setting and the direct contact to a health care professional such as the smoking cessation counselor. Alternatively, web-mode treatment might be better suited for externally motivated patients because they already arrive at the treatment with a default desire to do what they are told and are therefore more likely to stick to the web mode.

Furthermore, the differences in the blended treatment adherence levels are noteworthy. In web mode, the adherence level was in the expected range, whereas there was overadherence found for the face-to-face mode. This could be related to the fact that the treatment basically starts with a rather long face-to-face session and therefore results in a type of face-to-face default mode. Therefore, it is possible that the result would have been different if the treatment had started in web mode. The overadherence raises another question as to which level of adherence is optimal to reach the treatment goals; that is, is higher adherence (152% adherence to face-to-face mode) better than lower adherence (ie, 75% adherence to web mode)?

### Conclusion

This study represents one of the first attempts to thoroughly compare adherence and predictors of adherence of a blended smoking cessation treatment to a face-to-face treatment. Our results showed that the levels of adherence to both treatments were comparable. However, within the blended treatment, we found that adherence to face-to-face mode was significantly higher than that of web mode, although the intended total treatment time for the blended treatment was fairly broadly adhered to. This supports the idea that in blended treatment, one mode of delivery can compensate for the weaknesses of the other. Older age was found to be a common predictor of adherence to the treatments. However, within the blended treatment, adherence to each mode was predicted by different characteristics: adherence to the face-to-face mode was associated with demographic characteristics only, whereas adherence to the web mode of the blended treatment was also associated with several smoking-related and health-related characteristics. This may indicate that these characteristics should be taken into account when designing a blended treatment. However, the finding that only a small amount of the variance could be determined by the characteristics examined in this study suggests that provider-related health system factors and time-varying patient characteristics can also play an important role and should be explored in future research.

## Appendix

Multimedia Appendix 1 Screenshots of the web sessions of the blended smoking cessation treatment.

Multimedia Appendix 2 CONSORT-eHEALTH checklist (V 1.6.1).

## Abbreviations

DASS: Depression Anxiety Stress Scale
eHealth: electronic health
MAPHSS: Maudsley Addiction Profile Health Symptoms Scale
RCT: randomized controlled trial
